# The Medium Composition Impacts *Staphylococcus aureus* Biofilm Formation and Susceptibility to Antibiotics Applied in the Treatment of Bone Infections

**DOI:** 10.3390/ijms231911564

**Published:** 2022-09-30

**Authors:** Justyna Paleczny, Malwina Brożyna, Ruth Dudek-Wicher, Karolina Dydak, Monika Oleksy-Wawrzyniak, Marcin Madziała, Marzenna Bartoszewicz, Adam Junka

**Affiliations:** 1 Department of Pharmaceutical Microbiology and Parasitology, Faculty of Pharmacy, Wroclaw Medical University, 50-556 Wroclaw, Poland; 2 Faculty of Medicine, Lazarski University, 02-662 Warsaw, Poland

**Keywords:** *Staphylococcus*, biofilm, bone, gentamycin, ciprofloxacin, levofloxacin, vancomycin, osteomyelitis

## Abstract

The biofilm-associated infections of bones are life-threatening diseases, requiring application of dedicated antibiotics in order to counteract the tissue damage and spread of microorganisms. The in vitro analyses on biofilm formation and susceptibility to antibiotics are frequently carried out using methods that do not reflect conditions at the site of infection. To evaluate the influence of nutrient accessibility on *Staphylococcus aureus* biofilm development in vitro, a cohesive set of analyses in three different compositional media was performed. Next, the efficacy of four antibiotics used in bone infection treatment, including gentamycin, ciprofloxacin, levofloxacin, and vancomycin, against staphylococcal biofilm, was also assessed. The results show a significant reduction in the ability of biofilm to grow in a medium containing elements occurring in the serum, which also translated into the diversified changes in the efficacy of used antibiotics, compared to the setting in which conventional media were applied. The differences indicate the need for implementation of adequate in vitro models that closely mimic the infection site. The results of the present research may be considered an essential step toward the development of in vitro analyses aiming to accurately indicate the most suitable antibiotic to be applied against biofilm-related infections of bones.

## 1. Introduction

The bacterial bone infection may develop due to direct transmission of pathogens from blood, wounds, surgical contamination, and trauma, or indirectly, due to the presence of such factors as, e.g., neuropathy or vascular insufficiency. The infection incidence leads to prolonged hospitalization, high-cost care, and increased level of pain and mortality rate. The frequency of occurrence of infection after orthopedic procedure ranges from 0.1% to 30% [1,2]. Advanced age, intravenous drug use, implant presence, diabetes, and immunodeficiency, are considered the factors increasing the risk of bone infection [3].

The *Staphylococcus aureus* is a leading cause of bone fracture-related infections, prosthetic joint infections, and bacterial osteomyelitis [2,4]. The high virulence of this bacteria is driven by the production of toxins and exoproteins, which inhibit osteoblast function, leading to their death and osteoclastogenesis [5,6,7]. The numerous proteins present on the staphylococcal cell surface (including protein A, bone sialoprotein, collagen adhesion protein, and fibronectin-binding proteins A and B), allow bacteria to adhere to the bone surface [8,9,10]. Having attached, staphylococci start to form a biofilm, which accelerates the development of the infection process. The biofilm is a multi-cellular, highly aggregated microbial society embedded within the self-produced extracellular matrix (ECM). The ECM main components include polysaccharides, proteins, glycoproteins, and extracellular DNA. The primary role of these compounds is maintaining the integrity of cells’ community, improving mechanical stability, provision of favorable micro-environment, and providing protection from immune cells and bactericidal agents [11]. The ECM composition depends not only on the specific biofilm-forming species, but also on the accessibility of nutrients and the environmental conditions in which the biofilm develops [11,12]. The numerous in vitro research proved that bacterial biofilms are more resistant to antibiotics than non-adhered bacteria, resulting from decreased diffusion of antimicrobial agents through the matrix and diversified metabolic phenotype of biofilm-forming cells [13]. Moreover, the highly cross-linked structure of the biofilm matrix favors the exchange of genes encoding virulence factors and determining resistance to antibiotics [14].

The most frequent (and dangerous, from the clinical point of view) antibiotic resistance mechanism of staphylococci is referred to as the methicillin resistance. The strains of *Staphylococcus aureus* displaying this mechanism are described as the MRSA (methicillin-resistant *Staphylococcus aureus*). The MR mechanism determines resistance to all β-lactams, including penicillins, cephalosporins, and carbapenems. The MR mechanism is encoded by *mecA* and *mecC* genes, which are located on a mobile staphylococcal chromosomal cassette. The MRSA strains are responsible for up to 28% of all cases of bone infection caused by *S. aureus* [15,16,17]. The presence of staphylococcal biofilm on bone tissue is of strictly detrimental character. It is due to virulence factors produced by bacteria and host inflammatory mediators causing bone resorption and pathogenic fractures [18]. The final stage of biofilm development is a dissociation of bacterial cells from the biofilm structure, followed by bacterial dispersion to the adjacent locations [19]. The high ability of staphylococci to form a biofilm and to resist antibiotic activity is the reason of ongoing search for new counter-measures, including application of nano-silver [20] or plant-based products [21], to name just a few of them. Nevertheless, until the new approaches are introduced, the routine therapy of bone infections involves invasive surgical procedures and prolonged antibiotic therapy. The antibiotic applied should display the high penetration to the bone tissue and sufficient antimicrobial activity. Nevertheless, systemic administration is associated with provision of the high doses of antibiotics penetrating internal organs, resulting in life-threatening side effects, such as kidney impairment, hypotension, as well as neutro- and thrombocytopenia [22,23,24,25]. To reduce the risk of complications, topical administration of antibiotics, which achieve the therapeutic concentration at the orthopedic infection sites, are recommended.

Gentamycin is one of the antibiotics used topically in bone infection and can be delivered through biodegradable or durable biocompatible carriers [26,27]. Gentamycin sulfate is also widely applied to treat methicillin-susceptible *S. aureus* bone infections. This aminoglycoside inhibits the biosynthesis of proteins through binding with a 30S ribosomal subunit. The bond causes premature termination of RNA translation, resulting in bacterial death. Bactericidal activity is reported against Gram-positive and Gram-negative bacteria, as well as against the biofilm communities formed by these pathogens [28,29].

Ciprofloxacin is an antibiotic of the fluoroquinolone drug class. It displays a bactericidal activity against Gram-positive and Gram-negative bacteria and is approved for bone infection treatment. Its mechanism of action relies on inhibiting the activity of DNA-gyrase and DNA-topoisomerase IV, which leads to DNA replication impairment. Consequently, bacterial divisions are inhibited, and cell lysis occurs [30].

Levofloxacin belongs to the fluoroquinolone group, and the mechanism of action is analogous to ciprofloxacin. The bactericidal activity was reported against Gram-positive, Gram-negative aerobes and atypical bacteria [31]. Levofloxacin is considered more effective regarding *S. aureus* than ciprofloxacin, and the efficiency of these two antibiotics may depend on the methicillin-resistance mechanism occurrence [32].

Vancomycin is the first-line antibiotic for MRSA-related infections [33]. The bactericidal action is exerted by interrupting the elongation of peptidoglycan, the main component of the bacterial cell wall. The vancomycin molecule binds with the peptidoglycan precursor, leading to its conformational alteration, thereby inducing cell wall decomposition and bacterial lysis [34]. Noteworthy, vancomycin displays a bactericidal effect against bacterial cells also within osteoblasts [35]. Fluoroquinolones and vancomycin are recommended antibiotics in the treatment of *S. aureus*-related infections. The latter antibiotic is reserved only for MRSA-related bone infections [33,36].

The European Committee on Antimicrobial Susceptibility Testing recommends performing the antibiotic susceptibility testing of bacteria in standard microbiological media [37]. Likewise, the numerous in vitro research on antimicrobial efficacy is carried out in the experimental settings and performed in the conventional microbiological broths. The molecular composition of these media is rich in polysaccharides, peptides, amino acids, and glucose, which in turn are scantily present at the site of bone infection [38].

Therefore, the aim of this research was to evaluate the ability of methicillin-susceptible and methicillin-resistant *Staphylococcus aureus* strains, isolated from bone infections, to form biofilm in various culturing media. Three media were applied: tryptic soy broth, tryptic soy broth supplemented with 1% glucose and Dulbecco’s Modified Eagle’s Medium with 10% fetal bovine serum. Subsequently, antimicrobial and antibiofilm efficiencies of four antibiotics: gentamicin, ciprofloxacin, levofloxacin, and vancomycin in these media, were compared. Tryptic soy broth is a standard microbiological medium rich in proteins and glucose, commonly used in experiments assessing bacterial ability to form biofilm and their susceptibility to antimicrobials [39,40]. To assess the influence of glucose concentration on bacterial growth, we applied tryptic soy broth supplemented with 1% glucose, bearing in mind the high glucose concentration in blood of diabetic patients. Contrary to tryptic soy broth, Dulbecco’s Modified Eagle’s Medium is mainly composed of amino acids, vitamins and inorganic salts. These components are commonly present in serum and exudate [41,42]. Fetal bovine serum was added to supply the medium with compounds naturally occurring in living organisms. Therefore we speculate that Dulbecco’s Modified Eagle’s Medium with 10% fetal bovine serum mimics infection site to the higher extent than standard microbiological media. This research is a development of our previous study, which aimed to assess the activity of wound antiseptics against biofilm formed in the different conditions [43]. The significant results we obtained induced us to extend our research to strains isolated from bone infection and compare their antibiotic susceptibility in various culture conditions.

## 2. Results

**Figure 1:**
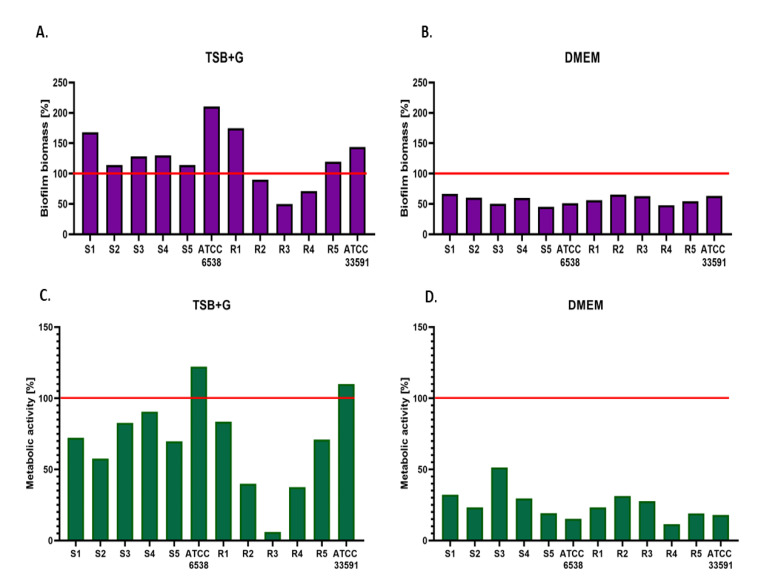
Comparison of the amount of biofilm biomass (**A,B**) and bacterial metabolic activity (**C**,**D**) between biofilms of methicillin-susceptible (S1–S5, ATCC 6538) and methicillin-resistant *Staphylococcus aureus* (R1–R5, ATCC 33591) formed in tryptic soy broth with 1% glucose (TSB + G) or Dulbecco’s Modified Eagle Medium (DMEM) with regard to results obtained for cultures in standard tryptic soy broth (TSB), which are expressed as 100% in the figure (red line); ATCC—American Type Culture Collection.

In the first line of the experiment, we assessed the ability of tested strains to form biofilm biomass (using the crystal violet method, CV) and the strains’ metabolic activity (using Richard’s method, RM). All strains were able to form biofilm on the surface of the 96-well plate. The results indicate that the amount of biomass produced by bacteria cultivated in TSB (Tryptic Soy Broth) was higher than in TSB + G (Tryptic Soy Broth + Glucose) (Figure 1, Table S1). Simultaneously, the metabolic activity of biofilm formed in TSB was lower than that observed for biofilms cultured in TSB + G. However, these differences were not statistically significant. In comparison, the amount of biomass assessed with crystal violet staining was significantly lower for strains cultivated in DMEM (Dulbecco’s Modified Eagle’s Medium) than in TSB and TSB + G (K-W, *p* < 0.001) (Figure 1, Table S1). The exact relation was observed for Richard’s method, which indicated that the metabolic activity of the strains grown in DMEM was significantly lower than in the other media applied (K-W, *p* < 0.001 for TSB, and *p* < 0.003 for TSB + G). The results for the biofilm cultures in DMEM were more coherent (of very low standard deviations) than the results obtained for the cultures in TSB and TSB + G. No statistically significant differences were obtained between ability of MSSA and MRSA to form biofilm in tested media.

Furthermore, the results show a positive correlation between the biomass amount and the metabolic activity for all strains cultured in TSB + G and DMEM (Table 1, Figure S1). The correlation was also statistically significant for MRSA and MSSA separately in the media mentioned above. The correlation coefficients were determined as very high for all strains cultured in TSB + G and for MRSA cultured in TSB + G; and high for all strains MRSA cultured in DMEM and MSSA cultured in TSB + G. An average correlation coefficient was obtained for MSSA cultured in DMEM. In the TSB medium, no statistically significant correlation was determined (Table 1, Figure S1).

The disk diffusion and microdilution methods were applied to scrutinize the antibiotic resistance of tested strains. Regarding VANCO, the antibiotic disc was replaced with a test strip containing a range of antibiotic concentrations. Antibiogram results show that 1 of 12 strains was resistant to GENTA, CIPRO, and LEVO concurrently (Table S2). Resistance to CIRPO and LEVO was shown in 2/12 strains. All *S. aureus* strains were susceptible to VANCO. The results of the disk diffusion method did not fully correlate with the results of the microdilution method for GENTA and VANCO (Table S3). The lowest concordance of results was determined for GENTA. In the case of the VANCO results, the concordance was obtained for 11/12 strains.

**Figure 2:**
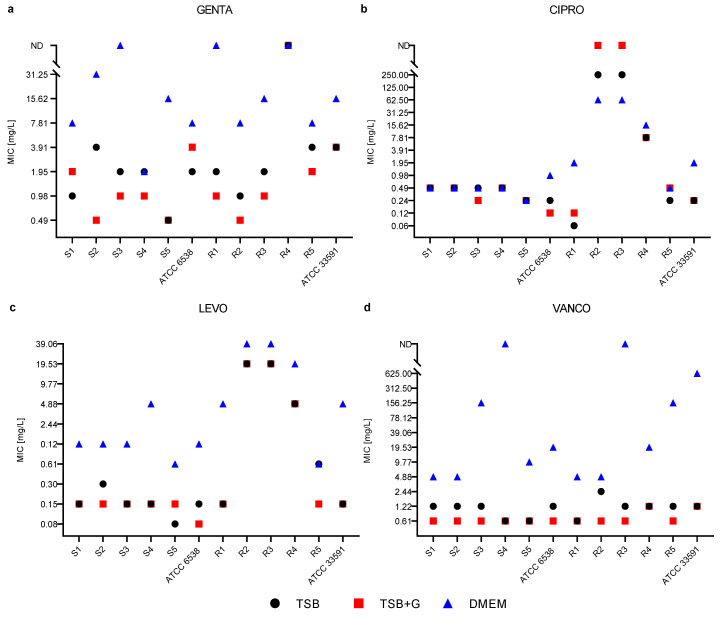
Minimal inhibitory concentrations MIC of gentamycin GENTA (**a**), ciprofloxacin CIRPO (**b**), levofloxacin LEVO (**c**), and vancomycin VANCO (**d**) of methicillin-susceptible S1–S5, methicillin-resistant R1–R5 and American Type Culture Collection ATCC *Staphylococcus aureus* strains cultivated in three media: tryptic soy broth TSB, tryptic soy broth with 1% glucose TSB + G and Dulbecco’s Modified Eagle Medium DMEM; ND—refers to the indeterminable minimal inhibitory concentration in the tested concentrations.

The microdilution method was applied to scrutinize the minimal inhibitory concentrations (MIC) of tested antibiotics against staphylococcal planktonic cells cultivated in various culture media. The results for specific strains cultivated in a particular medium are presented in Figure 2. Antibiotics were tested in a concentration range between 50–0.098% of their working solutions. MICs of GENTA and VANCO of two strains cultivated in DMEM and CIPRO for two strains cultivated in TSB + G were indeterminable because the antibiotics’ concentrations in the working solution were insufficient. According to the antibiogram results, these strains were susceptible to the aforementioned antibiotics (Table S2). The MIC values obtained in TSB and TSB + G were equal or differed by up to one serial dilution (Figure 2, Table 2), with an exception recorded for GENTA, where the difference was in range of three serial dilutions. The MIC values in DMEM were significantly higher than these obtained in TSB and TSB + G (Figure 2, Table 2) and differed up to nine serial dilutions (as recorded for VANCO). Interestingly, MIC values of CIPRO were comparable, regardless of the medium (Figure 2, Table 2). For 7/12 strains, the MIC was the same or differed up to one serial dilution. Moreover, high diversity in MICs of CIPRO and LEVO among tested strains was observed. For these antibiotics, half of MRSA strains were marked by higher MIC values than MSSA strains. In order to compare the influence of the culture medium on the clinical interpretation of the antibiotic resistance result, the antibiograms were compared with the MIC values obtained by the disc diffusion method. The concordance of clinical interpretations (susceptible or resistant) determined with the two methods for TSB and TSB + G cultures was 68.19%, while for DMEM cultures, the concordance was lower at 54.55%.

**Figure 3:**
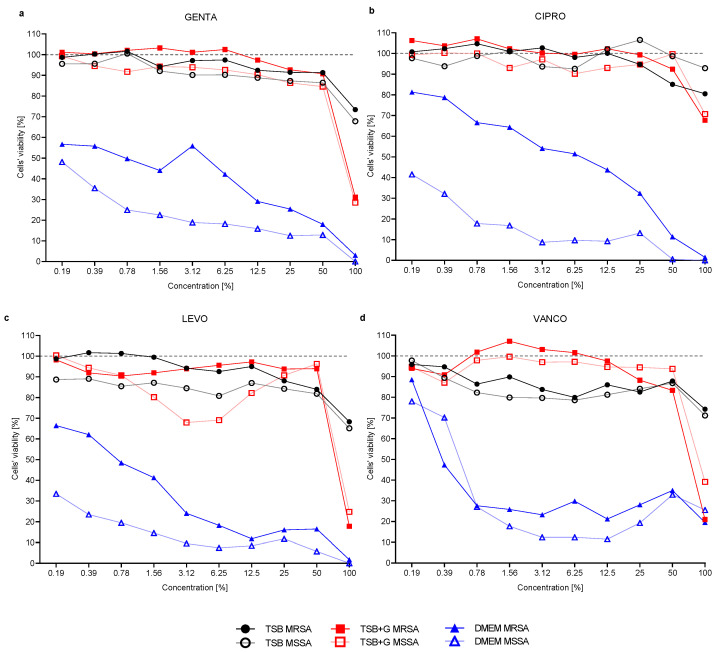
Viability of methicillin-susceptible MSSA and methicillin-resistant MRSA *Staphylococcus aureus* strains in biofilm, treated with serial dilution of antibiotic solutions: gentamycin GENTA (**a**), ciprofloxacin CIPRO (**b**), levofloxacin LEVO (**c**), and vancomycin VANCO (**d**). Biofilm was formed in three different media: tryptic soy broth TSB, tryptic soy broth with 1% glucose TSB + G, and Dulbecco’s Modified Eagle Medium DMEM.

In the next stage of the experiment, we determined the minimal biofilm eradication concentration (MBEC) of antibiotics. The highest concentration of antibiotics applied in the experiment was equal to 100% of the working solutions, precisely for GENTA and CIPRO, it was 2 g/L, and for LEVO and VANCO, it was 5 g/L. However, even such high concentrations of antibiotics were not sufficient to completely eradicate the biofilm formed on the microtiter plate surface (Table S4), so the MBEC values were undeterminable. Hence, we evaluated the drop in the viability of staphylococci cells in biofilm under exposure to various concentrations of antibiotics compared to the biofilm grown in culture media without antibiotic addition. The viability of MSSA and MRSA in the biofilm formed in TSB after exposure to 100% antibiotic solution was higher than the viability of biofilm formed in TSB + G and DMEM (Figure 3). The maximum concentration of GENTA, CIPRO, and LEVO reduced the viability of MSSA and MRSA cultured in DMEM by more than 90%. When the antibiotic concentration decreased, the viability of bacteria cultured in TSB and TSB + G was comparable. The lowest tested concentration of antibiotic-containing products was 0.19%, corresponding to the following antibiotic concentrations: for GENTA and CIPRO, it was 3.8 mg/L, and for LEVO and VANCO, it was 7.6 mg/L. Such low concentrations of antibiotics did not affect the viability of staphylococci cells in the biofilm formed in TSB and TSB + G. However, such low antibiotic concentrations were sufficient to reduce the viability of cells in biofilm grown in DMEM by up to 77% (MSSA, LEVO). Moreover, for each of the antibiotics, we observed higher viability of MRSA in comparison to MSSA in the given concentrations of antibiotics (Figure 3).

**Figure 4:**
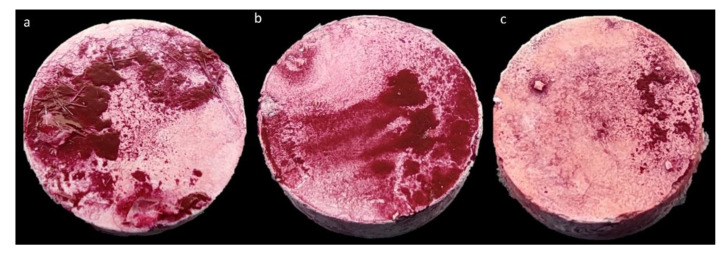
Biofilm of *Staphylococcus aureus* ATCC 6538 formed on the hydroxyapatite disc in three culture media: a tryptic soy broth TSB (**a**), tryptic soy broth with 1% glucose TSB + G (**b**) and Dulbecco’s Modified Eagle Medium DMEM (**c**). The biofilm is stained red with use of TTC compound.

Finally, we performed the modified antibiofilm dressing’s activity measurement assay (A.D.A.M.) to assess the ability of tested antibiotics to eradicate biofilm (Figure 4). The method was performed in TSB on all tested strains. Next, based on the results obtained, the most and the least resistant strain to each antibiotic and reference strains were chosen for further analysis in TSB + G and DMEM.

**Figure 5:**
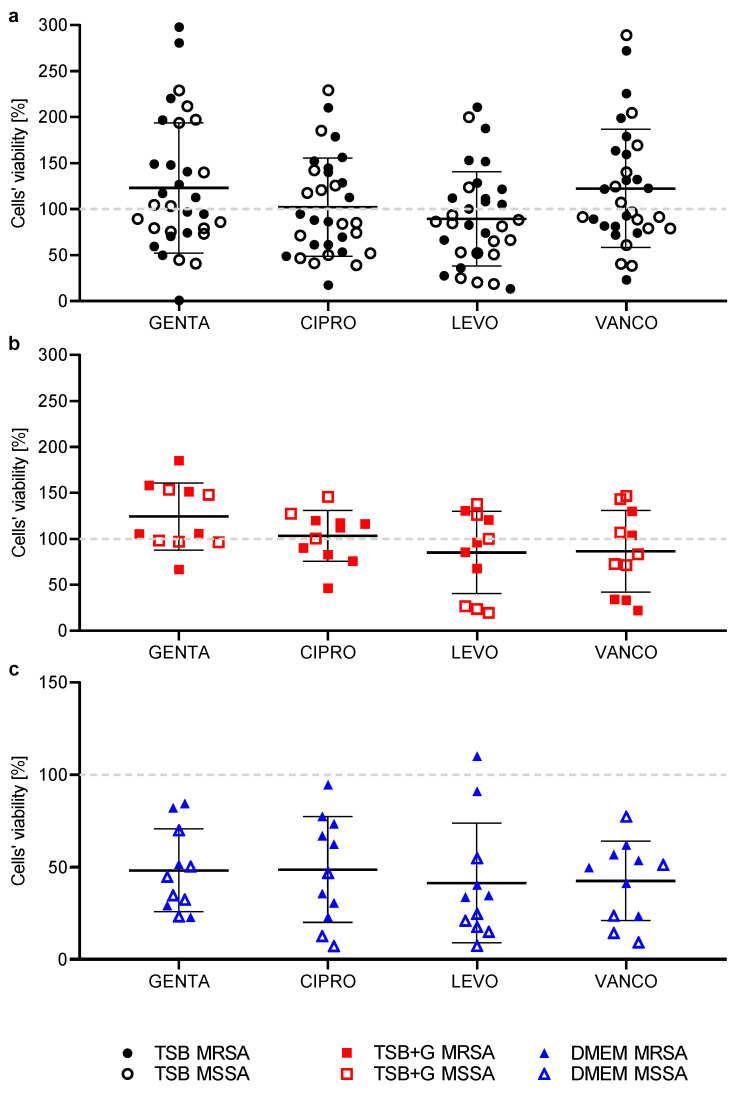
Viability of methicillin-susceptible MSSA and methicillin-resistant MRSA *Staphylococcus aureus* cells in biofilm formed on the hydroxyapatite discs in three different culture media: (**a**) tryptic soy broth TSB, (**b**) tryptic soy broth with 1% glucose TSB + G, and (**c**) Dulbecco’s Modified Eagle Medium DMEM, after exposure to gentamycin GENTA, ciprofloxacin CIPRO, levofloxacin LEVO, and vancomycin VANCO. Average and standard deviations were calculated for three replications obtained in one experiment, repeated two more times for all strains.

Results of the A.D.A.M. assay are presented in Figure 5 as a percentage of cells’ viability exposed to antibiotics compared to the unexposed cells, for which the metabolic activity was considered 100% (Figure 4). Moreover, results are presented for MSSA and MRSA strains separately, but the mean and standard deviation were calculated for all tested strains. The final concentrations of antibiotics in bacterial cellulose discs were as follows: GENTA and CIPRO 1.14 g/L, LEVO and VANCO 2.86 g/L. In the case of biofilm formed in TSB, LEVO was the only active antibiotic among the tested ones. The biofilm eradication activity of LEVO and VANCO against biofilm formed in TSB + G was comparable. GENTA and CIPRO did not perform any eradication activity in the case of the biofilm formed in TSB and TSB + G. However, all tested antibiotics showed activity against the biofilm formed in DMEM. The highest activity was observed for LEVO and VANCO (49% and 48% reduction rate, respectively). In the case of CIPRO and GENTA, the decrease in cells’ viability was comparable and reached 42%. No significant differences were determined between antibiotic activity in each medium.

## 3. Discussion

The bone infection is mainly caused by bacteria displaying the ability to form biofilm on biotic (bone) and abiotic (bone implant) surfaces. This highly-organized and cross-linked structure hinders the penetration of drugs to bacterial cells and allows pathogens to infect adjacent tissues and to rapidly expand the infection area. The biofilm-forming bacterial cells display diversified metabolic activity and the possibility of fast switching from a dormant, slow-growing cellular phenotype, to a phenotype displaying high metabolic activity. It contributes to the biofilm’s increased tolerability to antimicrobials. The majority of studies carried out on pathogens isolated from different sites of infection are conducted in ex vivo or in vitro settings, using conventional microbiological media. Whether—and to what extent—the results obtained by these methods translate to the phenomena, factually occurring in the living organism, remains unclear. As mentioned earlier, environmental conditions determine the structural and chemical composition of the biofilm matrix and the metabolic activity of biofilm-forming cells [12]. Therefore, it is essential to mimic the infection site environment to the highest extent possible in order to analyze the efficacy of an antibiotic in biofilm eradication. Presently, the increasing number of research is being carried out to develop the accurate models and to provide the answer on the factual activity of antibiotics in the site of infection [44,45].

**Figure 6:**
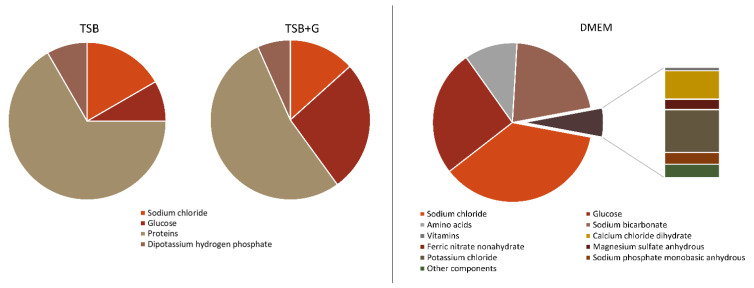
The percentage compositions of three media: tryptic soy broth TSB, tryptic soy broth with 1% glucose TSB + G, and Dulbecco’s Modified Eagle Medium DMEM.

The liquid milieu surrounding biofilm is one of the key variables in the process mentioned. In this work, various media were analyzed in order to assess their impact on bacterial (including biofilm) viability and antibiotic susceptibility (Figure 6). Tryptic soy broth (TSB) was applied as a standard microbiological broth, while TSB supplemented with glucose (TSB + G) was applied to determine the influence of glucose on bacterial growth. In turn, Dulbecco’s Modified Eagle Medium (DMEM) is considered to provide optimal conditions for the growth of bone cells referred to as the osteoblasts. Determining the exact conditions at the infection site is highly challenging due to the difficulty of collecting material. Nevertheless, it may be assumed that the components in DMEM reflect these conditions to a higher extent than TSB, thanks to elements naturally occurring in human serum. The addition of fetal bovine serum (FBS) allows for providing of all compounds present in serum that are lacking in DMEM.

The glucose concentration in all three media differs and is as follows: TSB—2.5 g/L, TSB + G—10 g/L, and DMEM—4.5 g/L. The obtained data indicate no statistical differences between biofilm amount and metabolic activity between cultures in TSB and TSB + G (Figure 1, Table S1). The results stay in line with our previously published data [43]. Furthermore, Miao et al., tested the influence of glucose on *S. aureus* biofilm formation in a wider range of concentrations (5–100 g/L) than was done in the present research [46]. It was noticed that the amount of biomass slightly increased during the time of incubation, but there were no statistically significant differences between cultures in these different concentrations of glucose. The metabolic activity of cultures (with the glucose concentration up to 30 g/L) was similar to the control with no glucose added. It was proven that during the bacterial glucose metabolism, the environment acidifies, which inhibits extracellular protease production. It enhances the binding of the biofilm matrix proteins to the cell surfaces and consequently stimulates biofilm formation [47]. Interestingly, Reffuveille et al., showed that the biofilm formation was increased in culture with no glucose added in comparison to the culture in which 4 g/L of glucose was provided [48]. However, this conclusion was drawn from analysis performed for only single staphylococci strain. It is worth noting that discrepancies between publications may be caused by the diverse glucose consumption as an energy source among tested strains displaying interspecies variability. Waldrop et al., determined a threshold for glucose concentration resulting in enhanced biomass amount [49]. They showed that 0.2 g/L and 1.6–2 g/L of glucose significantly affected the amount of biomass. Therefore, all media applied in the present work contained glucose in a concentration higher than the threshold level.

The ability to form biofilm was significantly lower in DMEM than in TSB and TSB + G. As mentioned above (and presented in Figure 6), DMEM composition significantly differs from TSB. Amino acids, such as arginine, cysteine, glutamine, histidine, methionine, glycine, phenylalanine, and tyrosine, are the main components of DMEM. These amino acids play an important role in bone development, but also regulate biofilm formation [50]. The inhibitory effect of tyrosine, phenylalanine, and cysteine relies on hindering bacteria from aggregating into large clusters, which inhibits biofilm development [51,52]. This suggests that specific amino acids may prevent the maturation of biofilms, facilitating their eradication. The calcium and bicarbonate ions are also present in high abundance in the bone environment [48]. Shukla et al., showed that the staphylococcal biofilm architecture varied in response to different concentrations of calcium [53]. As the concentration of calcium in the culture medium increased, total biofilm biomass and its density and thickness decreased. The conformational alterations may be indicated through calcium interactions with a clumping factor B (ClfB) and/or biofilm-associated protein (Bap) [54,55]. Sodium bicarbonate serves as a source of bicarbonate in DMEM in a concentration of 3.7 g/L. Previously published data show an inhibitory effect of bicarbonate sodium on *S. aureus* growth, caused by alternations in thickness of bacterial cell wall and membrane thickness [56]. The minimal inhibitory concentrations were as follows: 2.1 g/L, 10.5 g/L, and 21 g/L [57,58,59]. The data discrepancies may be due to the different media compositions in which the strains were cultured and the intraspecies variations. However, no antibiofilm effect was previously described. The influence of specific components on bacterial growth explains decreased biofilm formation in DMEM and more robust biofilm development in TSB, devoid of these components. Additionally, the results obtained for cultures in DMEM were more coherent than these obtained for cultures in TSB and TSB + G. It indicates that *S. aureus* strains metabolize given nutrients on a similar level, so the results obtained for cultures in DMEM are of higher repeatability. The half of the tested *S. aureus* strains were methicillin-resistant. The biofilm biomass amount and metabolic activity of MRSA were similar to MSSA (Figure 1, Table S1), and are consistent with previous results, which indicate no relationship between methicillin-resistant mechanism and biofilm-formation [60]. The results support the claim that the glucose-induced mechanism of biofilm formation in MRSA is impaired by the mutation, which upregulates the protease activity [61].

Additionally, the correlations between the amount of biofilm biomass and the metabolic activity of cells embedded in the structure were calculated (Table 1, Figure S1). No linear correlation was determined for *S. aureus* cultured in TSB. However, significant values were obtained for staphylococcal strains (also for MSSA and MRSA separately) cultured in TSB + G and DMEM, indicating positive correlations. The correlation may be induced by enhanced glycolysis resulting from higher sugar concentrations in TSB + G and DMEM than in TSB. Nevertheless, many variables impact metabolic activity, such as biofilm niches with decreased cell viability. It explains no correlation in TSB presented in our study, or the results of Xu et al., showing that 30% of staphylococcal strains displayed a negative correlation [62].

In the next stage of the study, the efficacy of antibiotics was evaluated using the disk diffusion and microdilution method in three culture media (Tables S2 and S3). MICs values of GENTA, LEVO, and VANCO, determined in DMEM, differed significantly from values obtained in TSB and TSB + G (Figure 2, Table 2). In the case of CIPRO, the MIC values largely concur with those obtained in other media. The differences in MIC values obtained between DMEM and other media indicate a considerable influence of the culture medium on the inhibitory activity of antibiotics. It is reported that different media compositions affect antibiotic activity [63,64]. We assume that nutrients provided in culture media not only impact the bacterial metabolic activity but also interfere with the antimicrobial agents, changing their efficacy. Nanavaty et al. reported that MIC of aminoglycosides was increased even 8-fold in response to higher calcium and magnesium concentrations [65]. Additionally, the lower pH contributed to the decreased activity of the aminoglycosides, which can be induced by the altered degree of the compounds’ ionization [65]. In order to compare the influence of the culture medium on the clinical interpretation of the antibiotic resistance result, the antibiograms were compared with the MIC values obtained by the disc diffusion method. The concordance of clinical interpretations determined with these two methods for TSB and TSB + G cultures was 68.19%, while for DMEM cultures, the concordance was lower at 54.55% (Tables S2 and S3). The problem was also addressed by Walsh et al. They demonstrated the very low sensitivity of microdilution assays performed in CaMHB and BHI for vancomycin, compared to the strip diffusion test for higher sensitivity and gently lower specificity [66]. The differences in MIC values obtained in disk diffusion and the microdilution method may result in inconsistency in clinical outcomes, which is a critical issue for treatment decisions.

The efficacy of antibiotics in biofilm eradication was also evaluated. The total biofilm eradication and determination of MBEC values were possible only for several strains cultured in DMEM (Table S4). This stays in line with the well-described observation that MIC values are mostly lower than these of MBEC, resulting, among others, from impaired penetration of the drug [67]. Our results indicate that the lower ability of bacteria to form biofilm in DMEM than in TSB or TSB + G translates into more effective eradication. As presented in Figure 3, even low concentrations of antibiotics decreased the bacterial viability in biofilm formed in DMEM at a higher level than in biofilm formed in TSB and TSB + G. Interestingly, GENTA, CIPRO, and LEVO reduced cells’ viability in biofilms in DMEM in varying levels depending on the methicillin resistance. The phenomenon was not observed for biofilms formed in TSB and TSB + G. The influence of media on the biofilm removal was also described by Chen et al., who compared eradication of *S. aureus* biofilm cultured in TSB and CaMHB (cation-adjusted Mueller Hinton) with vancomycin. The diverse composition of media influenced the vancomycin efficacy, indicating that biofilm formed in TSB was more difficult to remove [68].

**Figure 7:**
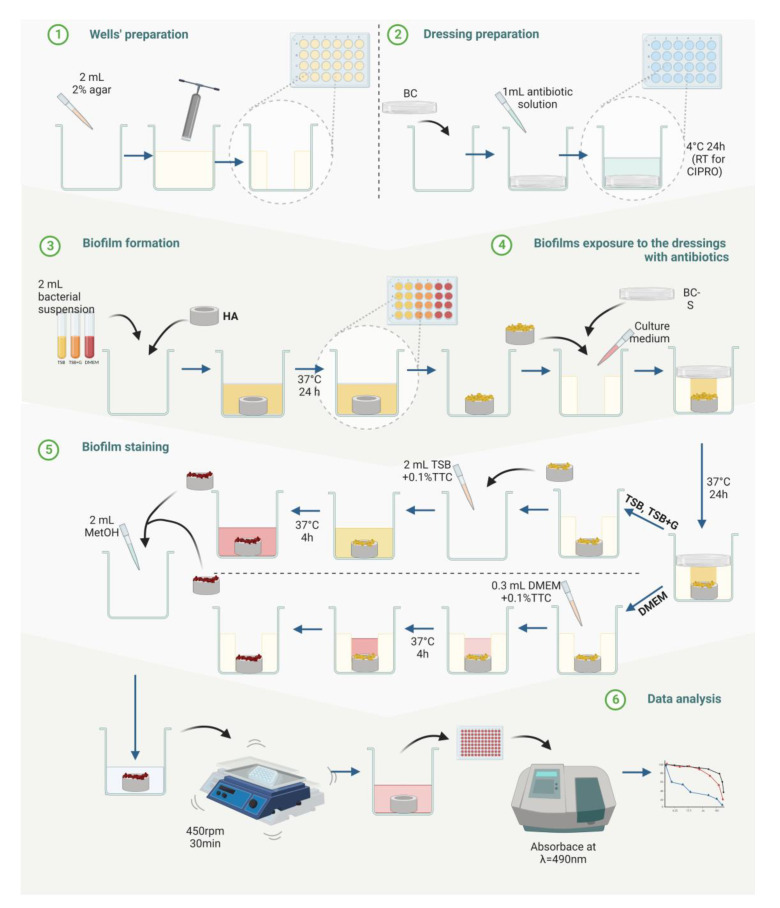
Schematic diagram of modified antibiofilm dressing’s activity measurement (A.D.A.M.). BC—bacterial cellulose, BC-S—bacterial cellulose saturated with antibiotic, HA—hydroxyapatite disk, TSB—tryptic soy broth, TSB + G—tryptic soy broth with 1% glucose, DMEM—Dulbecco’s Modified Eagle’s Medium, TTC—tetrazolium chloride solution, and MetOH—methanol.

In the study’s final stage, the efficacy of antibiotics in biofilm eradication was evaluated using the modified antibiofilm dressing’s activity measurement assay (A.D.A.M.). This method was developed by our team to consider multiple factors to reflect the site of infection [69,70] (Figure 7). The biofilm is formed on a rough hydroxyapatite surface that is the main inorganic component of a bone (Figure 4). The antibiotic is diluted in the cellulose carrier and in the culture medium added to the setup, similar to the infection site where inflammatory and purulent exudate is produced and impedes the antibiotic penetration. The results obtained with this method show significant differences in biofilm eradication efficiency by antibiotics, depending on the culture medium in which bacteria were cultivated (Figure 5). Most of the tested strains cultured in TSB and TSB + G showed low sensitivity to the applied antibiotics or even increased metabolic activity compared to the growth control. Significant differences were also observed between the results for individual strains; for cultures in TSB, these differences were as high as 250%. On the other hand, eradication of biofilm formed in DMEM was about 50% for all antibiotics used. These results correlate with those we obtained regarding the biofilm-forming ability of bacteria and determining MBEC. The lower biofilm-forming ability of the bacteria translated into higher eradication of biofilm formed even on a very rough surface.

The reported data confirm the high importance of adjusting the conditions for microbial culture in in vitro studies on the efficacy of antimicrobial substances. Therefore, much research is carried out on the biofilm growth model to effectively mimic the conditions at the site of the bone infection [44,45]. The use of hydroxyapatite disks made it possible to examine biofilms on bone-like material. The nutrients provided by the DMEM with FBS are more similar to substances present at the site of infection than those provided by basic microbial media. Using native bacterial cellulose enables us to avoid the antibiotic’s interferences with the drug carrier. We are convinced that the data presented in the paper, showing differences in the efficacy of antibiotics depending on the culture medium, will allow for paving the way for improved methods aiming to reflect the environment of the infection site, and by provision of the reliable data with this regard, will eventually allow for the prediction of the factual activity of antibiotics against staphylococcal biofilm.

## 4. Materials and Methods

### 4.1. Tested Microorganisms

The research was performed on two reference strains from the American Type Culture Collection (ATCC), such as *Staphylococcus aureus* 6538 (methicillin-susceptible *Staphylococcus aureus* MSSA) 33591 (methicillin-resistant *Staphylococcus aureus* MRSA), and 10 strains isolated from bone infections. Clinical strains included MSSA (n = 6) and MRSA (n = 6). All 12 tested strains originate from the Strains Collection of The Department of Pharmaceutical Microbiology and Parasitology, Medical University of Wroclaw, Poland.

### 4.2. Culture Conditions

Bacteria were cultured in three media:

1. Tryptic Soy Broth (Biomaxima, Lublin, Poland), labeled as TSB;
2. Tryptic Soy Broth (Biomaxima, Lublin, Poland) with 1% of glucose supplementation (*w*/*v*; Chempur, Piekary Slaskie, Poland), labeled as TSB + G;
3. Dulbecco’s Modified Eagle’s Medium High Glucose (Biowest, Riverside, MO, USA; cat no. L0103) supplemented with 10% of fetal bovine serum (Biowest, Riverside, MO, USA), labeled as DMEM.

### 4.3. Tested Antibiotics

The following four antibiotics were chosen for the experimental purposes:

1. Gentamycin as gentamycin sulfate, powder for the solution of final concentration 2 g/L (Pol-Aura, Dywity, Poland), labeled as GENTA;
2. Ciprofloxacin as monohydrate ciprofloxacin hydrochloride, solution for infusion in concentration 2 g/L of the active substance (Cipronex^®^) (Polpharma, Starogard Gdanski, Poland), labeled as CIPRO;
3. Levofloxacin as hemihydrate levofloxacin; solution for infusion of 5 g/L active substance (Levofloxacin Kabi^®^) (Fresenius Kabi, Warsaw, Poland), labeled as LEVO;
4. Vancomycin as vancomycin hydrochloride; powder for infusion solution of final concentration 5 g/L (Vancomycin-MIP^®^) (MIP Pharma, Gdansk, Poland), labeled as VANCO.

### 4.4. Evaluation of Biofilm Biomass Level Using the Crystal Violet Dying Method [CV] in 96-Well Microtiter Plate

Bacterial inoculum at a density of 0.5 McF (McFarland turbidity scale) (established using densitometer Densitomat II, BioMerieux, Warsaw, Poland) was prepared from the 24 h planktonic cultures in TSB, TSB + G, and DMEM. The inoculum was diluted to obtain ca. 1 × 10^5^ CFU/mL (colony-forming unit, CFU) and added to the six wells in the 96-well microtiter plate (VWR, Radnor, PA, USA), with 100 μL to each well. The plate was incubated for 24 h at 37 °C under static conditions. After a time, the medium above the biofilm was discarded, and the plate was moved for 10 min drying at 37 °C. Next, 100 μL of 20% water solution of crystal violet (*v*/*v*) (Aqua-med, Lodz, Poland) was poured into each well and incubated for 10 min at room temperature. Next, the solution was discarded, and the attached biofilm was gently rinsed twice with 100 μL of 0.9% NaCl (Chempur, Piekary Slaskie, Poland). The plate was dried for 10 min at 37 °C, and afterward, 100 μL of 30% aqueous solution of acetic acid (*v*/*v*) (Chempur, Piekary Slaskie, Poland) was added to the wells. The plate was set to a mechanical shaking at 450 rpm for 30 min (Schuttler MTS-4, IKA, Königswinter, Germany). Next, the colour solution was transferred to a new plate to measure the absorbance of the extracted CV at a 550 nm wavelength (MultiScan Go Spectrophotometer, Thermo Fischer Scientific, Waltham, MA, USA). The crystal violet staining method was performed in six repetitions in three independent experiments for each strain.

### 4.5. Evaluation of Biofilm Metabolic Activity Using Richard’s Method [RM] in 96-Well Microtiter Plate

The initial stage of the method, involving the biofilm inoculation in the wells of 96-well microtiter plate wells, was carried out as described in Section 4.1. After that, the culture medium was gently removed from the wells. The 0.1% tetrazolium chloride solution (2,3,5-triphenyl-2H-tetrazolium chloride, TTC) (PanReac AppliChem, Darmstadt, Germany) was prepared in the tested media: TSB, TSB + G, and DMEM. Next, 100 μL of such solution was added to each well of the 96-well microtiter plate (VWR, Radnor, PA, USA) with adhered biofilm and incubated for 2 h at 37 °C. Colorless TTC is transformed into red formazan crystals by metabolically active cells. Afterward, 100 μL of methanol (Chempur, Piekary Slaskie, Poland) was poured into each well to dissolve the formed red crystals. The plate was shaken at 450 rpm for 30 min (Schuttler MTS-4, IKA, Königswinter, Germany). Next, the solution was transferred into the new plate to measure the absorbance at a 490 nm wavelength (MultiScan Go Spectrophotometer, Thermo Fischer Scientific, Waltham, MA, USA). Richard’s method was conducted in three independent experiments of six replications for each strain.

### 4.6. Evaluation of Antimicrobial Susceptibility of Antibiotics Using Disc and Strip Diffusion Methods

Bacteria susceptibility to gentamycin, levofloxacin, and ciprofloxacin was assessed by a disc diffusion method. The test strip diffusion method was applied to assess the susceptibility to vancomycin. Bacterial suspension at a density of 0.5 McF (densitometer Densitomat II, BioMerieux, Warsaw, Poland) in sterile 0.9% NaCl (Chempur, Piekary Slaskie, Poland) was prepared from the 24 h cultures on Columbia agar plates (Becton, Dickinson and Company, Heidelberg, Germany). The inoculum was transferred on the Mueller–Hinton agar (Biomaxima, Lublin, Poland) using a sterile swab stick and inoculated with a test strain by streaking the swab three times over the entire agar surface. Disks impregnated with 10 µg gentamycin, 5 µg ciprofloxacin (purchased from Becton, Dickinson and Company, Sparks, MD, USA), and 5 µg levofloxacin (purchased from Oxoid Ltd., Basingstoke, UK), were placed on the inoculated agar plates at the minimal distance of 30 mm. The agar plates were incubated for 18 h at 35 °C. The test strips impregnated with a wide range of vancomycin concentrations (0.016–256 mg/L) (purchased from Liofilchem srl, Roseto degli Abruzzi, Teramo, Italy) were carefully placed on the inoculated agar plate. The incubation took 24 h at 35 °C. After incubation, the inhibition zones were measured, and minimal inhibitory concentrations were read and interpreted with the European Committee on Antimicrobial Susceptibility Testing breaking points table [71].

### 4.7. Evaluation of Minimal Inhibitory Concentration [MIC] of Antibiotics Using Spectrophotometric Assessment and Richard’s Method in 96-Well Microtiter Plate

The MICs of antibiotics were determined by the standard serial microdilution method in TSB, TSB + G, and DMEM in a 96-well microtiter plate (VWR, Radnor, PA, USA). The 24-h planktonic cultures in a specific medium were used for experimental purposes. Bacterial suspension at a density of 0.5 McF (densitometer Densitomat II, BioMerieux, Warsaw, Poland) was diluted to 1 × 10^5^ CFU/mL, and 100 μL of such prepared inoculum was added to wells within the antibiotics concentrations range. The highest concentrations of antibiotics were the following: GENTA 1 g/L, CIPRO 1 g/L, LEVO 2.5 g/L, and VANCO 2.5 g/L in the final stage. The control of bacterial growth (without antibiotics) and sterility control (without bacteria and antibiotics) were done. Next, the absorbance was measured at a 580 nm wavelength (MultiScan Go Spectrophotometer, Thermo Fischer Scientific, Waltham, MA, USA). The plate was transferred on a shaker (Schuttler MTS-4, IKA, Königswinter, Germany) and incubated for 24 h at 37 °C with a shaking frequency of 400 rpm. Subsequently, the absorbance was measured again. No difference between the absorbance values acquired at 0 and 24 h would determine an antibiotic concentration corresponding to the MIC value. Additionally, 20 μL of 1% tetrazolium chloride salt (2,3,5-triphenyl-2H-tetrazolium chloride) (PanReac AppliChem, Darmstadt, Germany) was added to wells on the microtiter plate and incubated at 37 °C to visualize metabolically active cells. The MIC values were determined within 2 and 24 h of the incubation as a concentration of a specific antibiotic in a first colorless well. Two replicates were conducted in each of three independent experiments of MIC evaluation.

### 4.8. Evaluation of Minimal Biofilm Eradication Concentration [MBEC] of Antibiotics Using Spectrophotometric Assessment and Richard’s Method in 96-Well Microtiter Plate

The standard serial microdilution method was also applied to determine the MBECs of antibiotics. The 24 h bacterial suspensions in TSB, TSB + G, and DMEM were used to obtain the inoculum at a density of 0.5 McF (densitometer Densitomat II, BioMerieux, Warsaw, Poland) and subsequently diluted to 1 × 10^5^ CFU/mL. An amount of 100 μL of such prepared suspension was added to the 96-wells microtiter plate (VWR, Radnor, PA, USA) and supplied with 100 μL of specific culture medium. The plate was incubated under static conditions for 24 h at 37 °C for biofilm development. Serial dilutions of antibiotics were freshly prepared in a specific culture medium with the highest concentrations following: GENTA 2 g/L, CIPRO 2 g/L, LEVO 5 g/L, and VANCO 5 g/L. After incubation time, non-adhered cells were removed, and the serial dilution of antibiotics was introduced to the biofilm at a volume of 200 μL to each well. The whole setting was incubated for the next 24 h at 37 °C under static conditions. The following day, the wells’ content was gently removed, and 200 μL of 0.1% tetrazolium chloride salt (PanReac AppliChem, Darmstadt, Germany) solution in a specific medium was added to the 96-well microtiter plate. The setting was incubated for 2 h at 37 °C. After a time, the MBEC was determined as a concentration of the antibiotic in the first colorless well, neighbored by the red one. The plate was repositioned at 37 °C for the next 22 h, and next, the medium was gently removed. Then, 200 μL of methanol (Chempur, Piekary Slaskie, Poland) was added into the wells and the plate was shaken for 30 min at 400 rpm (Schuttler MTS-4, IKA, Königswinter, Germany). Next, 100 μL of the solution was carried over to the new microtiter plate to measure its absorbance at a 490 nm wavelength (MultiScan Go Spectrophotometer, Thermo Fischer Scientific, Waltham, MA, USA). The growth and sterility controls, as well as a number of replicates, were performed as described in Section 4.7. Cell viability was calculated using the following Equation (1):

(1) $$\frac{{\mathrm{OD}}_{S}-{\mathrm{OD}}_{M}}{{\mathrm{OD}}_{\mathrm{GC}}-{\mathrm{OD}}_{M}}\;\times \;100\%$$

where OD_S_ refers to the absorbance value of samples treated with the antibiotic, OD_M_ refers to the methanol absorbance, and OD_GC_ refers to the absorbance values of growth control.

### 4.9. Evaluation of Biofilm Eradication from Bone Hydroxyapatite Using Modified Antibiofilm Dressing’s Activity Measurement [A.D.A.M.]

#### 4.9.1. Preparation of Bacterial Cellulose Dressings Chemisorbed with Antibiotics

*Komagataeibacter xylinus* (Deutsche Sammlung von Mikroorganismen und Zellkulturen—DSM 46602) culture was carried out in Herstin–Shramm medium [72] in a 24-well plate (VWR, Radnor, PA, USA) for 7 days at 28 °C under stationary conditions. After a time, bacterial cellulose (BC) was harvested from the medium and soaked in 0.1 M NaOH (Chempur, Piekary Slaskie, Poland) at 80 °C for 90 min to purify from culture residues. Next, BC was washed with distilled water repeatedly until neutral pH was obtained, sterilized in a steam autoclave, and stored at 4 °C. To prepare BC dressings chemisorbed with tested antibiotics (BC-S), sterile BC was soaked in 1 mL of specific antibiotic solution in a 24-well plate for 24 h at 4 °C (exceptionally, BC-S with ciprofloxacin was incubated at room temperature). The water capacity of the BC was estimated by weighing the dressings with the use of an analytical balance (Pioneer PA213CM/1, Ohaus, Switzerland) and then drying at 60 °C. The procedure was repeated until the weights stopped dropping. The concentration of the chemisorbed antibiotic was calculated from the Equation (2):

(2) $$\frac{M_{S}}{V_{\mathrm{BC}}+{\;V}_{S}}$$

where V_BC_ refers to the BC volume, V_S_ to the volume of substance added to the well, and M_S_ refers to the amount of the antibiotic in grams. Sterile BC was also soaked in 0.9% NaCl (Chempur, Piekary Slaskie, Poland) to serve as a control (BC-N) for further experiment stages.

#### 4.9.2. Preparation of Hydroxyapatite Discs Coated with Biofilm

The Hydroxyapatite powder pellets were pressed without binding to obtain HA discs of a diameter of 8 mm, sintered at 900 °C, and compressed for bending, compression, and static tensile tests using the universal testing system (Instron model 3384; Instron, Norwood). The manufactured hydroxyapatite discs (HA) mimicked the bone structure and were used as a surface for biofilm establishment and development. For this purpose, 24-h cultures of staphylococci strains in TSB, TSB + G, and DMEM were used. Bacterial suspension at a density of 1 McF (Densitomat II, BioMerieux, Warsaw, Poland) was diluted to obtain c.a. 1 × 10^3^ CFU/mL. Next, HA discs were placed into the wells in a 24-well plate (VWR, Radnor, PA, USA), and 2 mL of freshly prepared bacterial suspension was poured. The whole setting was incubated for 24 h at 37 °C in the stationary condition, and after a time, the medium was gently removed from the wells.

#### 4.9.3. Biofilm Eradication Activity of Chemisorbed Dressings

The modified A.D.A.M. assay was conducted according to the protocol devised by Krzyżek et al., and presented in Figure 7 [73]. For the experimental purpose, 2 mL of 2% agar (VWR Chemicals, Radnor, PA, USA) was poured into the wells of a 24-well plate (VWR, Radnor, PA, USA) and incubated at room temperature until solidification. Next, holes were cut in the middle of the agar using an 8 mm diameter cork borer. HA-coated with biofilm was transferred into the agar hole in such a way that the biofilm surface was at the top. The hollows were filled with approx. 300 μL with the specific culture medium to form a convex meniscus. Afterward, BC-S chemisorbed with tested antibiotics solutions were put on the top of the wells. BC-N was applied as growth control. The plate with the whole setting was covered with the lid and incubated under stationary conditions for 24 h at 37 °C. The following day, chemisorbed BCs (BC-S and BC-N) were discarded, the medium was gently removed, and HA was transferred to a new 24-well plate. To visualize metabolically active bacteria, 2 mL of 0.1% tetrazolium chloride solution (2,3,5-triphenyl-2H-tetrazolium chloride, TTC) (PanReac AppliChem, Darmstadt, Germany) freshly prepared in a specific medium was poured in the wells. After 4 h of incubation at 37 °C, the medium was removed, and 1 mL of methanol (Chempur, Piekary Slaskie, Poland) was added. The plate was subject to mechanical shaking at 450 rpm for 30 min (Schuttler MTS-4, IKA, Königswinter, Germany). After a time, the color solution was portioned to six wells in a 96-well microtiter plate (VWR, Radnor, PA, USA), 100 μL to each well, and measured spectrophotometrically at a 490 nm wavelength (MultiScan Go Spectrophotometer, Thermo Fischer Scientific, Waltham, MA, USA). The cells’ viability was calculated from the Equation (3):

(3) $$\frac{{\mathrm{OD}}_{\mathrm{BC}-S}-{\mathrm{OD}}_{M}}{{\mathrm{OD}}_{\mathrm{BC}-N}}\;\times \;100\%$$

where OD_BC-S_ refers to the absorbance value of samples treated with BC-S, OD_M_ refers to the methanol absorbance, and OD_BC-N_ refers to the absorbance values of growth control. The experiment was conducted in three independent experiments, triplicate for each antibiotic. All tested staphylococci cultured in TSB were primarily evaluated by the method. According to obtained results, we chose two strains for testing specific antibiotics (one the most susceptible, one the least susceptible to each antibiotic) in TSB + G and DMEM. Type strains were also included.

Two final stages of the method were modified for cultures in DMEM because of its insufficient growth on the HA discs. So, after BC-S and BC-N were discarded and the medium was gently removed, 300 μL of the 0.1% TTC was added into the agar holes. After the 4 h incubation at 37 °C, the solution was removed, and HA discs were placed into the fresh 24-well plate. Further stages were performed as described in the last paragraph. Briefly, 1 mL of methanol was poured into the wells with HA discs. The plate was shaken at 450 rpm for 30 min. Next, 100 μL of the color solution was transferred to 6 wells in the 96-well microtiter plate, and absorbance was measured at a wavelength 490 nm.

### 4.10. Statistical Analysis

Calculations were performed using GraphPad Prism (Version 8.0.1; GraphPad Software Inc., La Jolla, CA, USA). Normality distribution was assessed with the Shapiro–Wilk test, and variance homogeneity was assessed using the Brown–Forsythe test. The Pearson or Spearman correlation (depending on the distribution) was calculated to assess a linear correlation between biofilm biomass level and biofilm metabolic activity. Non-parametric ANOVA Kruskal–Wallis test with post-hoc Dunn’s analysis was performed to compare the efficacy of tested antibiotics. The results of statistical analyses with a significance level of *p* < 0.05 were considered significant.

## 5. Conclusions

- *Staphylococcus aureus* cultured in DMEM displayed lower biofilm-forming ability and decreased metabolic activity than biofilms cultured in TSB and TSB + G.
- The effectiveness of antibiotics in inhibiting growth and eradicating staphylococcal biofilm depends on the culture medium in which the bacteria are cultivated.
- It is necessary to conduct analyses on the efficacy of antibiotics in models that mimic the site of infection as closely as possible because of the large number of factors that influence their activity.

## Figures and Tables

**Table 1:** Results of correlations between the ability to form biofilm assessed with the crystal violet method (CV) and bacterial metabolic activity measured with Richard’s method (RM) calculated for all tested strains and separately for methicillin-susceptible *Staphylococcus aureus* MSSA and methicillin-resistant *Staphylococcus aureus* MRSA strains cultured in tryptic soy broth TSB, tryptic soy broth with 1% glucose TSB + G, and Dulbecco’s Modified Eagle Medium DMEM; *r*—correlation coefficient and *p*—probability level.

Crystal Violet vs. Richard’s Methods Correlations			
Tested Group	TSB	TSB + G	DMEM
**All strains**	*p* = 0.454	*p* < 0.0001	*p* < 0.0001
		*r* = 0.82	*r* = 0.61
**MRSA**	*p* = 0.307	*p* < 0.0001	*p* = 0.003
		*r* = 0.87	*r* = 0.66
**MSSA**	*p* = 0.951	*p* = 0.003	*p* = 0.027
		*r* = 0.66	*r* = 0.52

**Table 2:** Differences in minimal inhibitory concentrations MIC of gentamycin GENTA, ciprofloxacin CIPRO, levofloxacin LEVO and vancomycin VANCO between strains cultivated in three media: tryptic soy broth TSB, tryptic soy broth with 1% glucose TSB + G and Dulbecco’s Modified Eagle Medium DMEM. Arrow up indicates that the average MIC value in a particular medium was higher than in another medium (arrow down); *p*—probability level, for *p* < 0.05 differences were statistically significant and marked as (*) 0.033 and (***) < 0.001, while ns refers to differences being statistically insignificant.

Substance	Comparison of MIC Values in a Specific Medium								
	TSB vs. TSB + G	TSB	vs.	DMEM	*p*	TSB + G	vs.	DMEM	*p*
**GENTA**	ns	↓		↑	*	↓		↑	***
**CIPRO**	ns	ns		ns	ns	ns		ns	ns
**LEVO**	ns	↓		↑	*	↓		↑	*
**VANCO**	ns	↓		↑	*	↓		↑	***

## Data Availability

All necessary data are presented in the manuscript and in the Supplementary Materials and can be provided from the authors upon reasonable request.

## Supplementary Materials

The following supporting information can be downloaded at: https://www.mdpi.com/article/10.3390/ijms231911564/s1.
