# Supplementary material for: The Medium Composition Impacts Staphylococcus aureus Biofilm Formation and Susceptibility to Antibiotics Applied in the Treatment of Bone Infections
Source: Int J Mol Sci. 2022 Sep 30;23(19):11564. doi: 10.3390/ijms231911564 (PMC9569719; doi:10.3390/ijms231911564)
Supplement: Supplementary file 1 [file ijms-23-11564-s001.zip › ijms-1921795-supplementary.pdf]

Table S1. Ability to form biofilm assessed by Crystal Violet (**CV**) and Richard's (**RM**) methods of methicillin-susceptible (**MSSA**) and methicillin-resistant (**MRSA**) *Staphylococcus aureus* strains cultivated in three media: tryptic soy broth **TSB**, tryptic soy broth with 1% glucose **TSB+G** and Dulbecco's Modified Eagle Medium **DMEM** expressed as an average values of absorbance measured at 550 nm (CV) and 490 nm (RM) wavelenght and standard deviations in brackets.

Method	Strain	Absorbance					
		TSB		TSB+G		DMEM	
CV	ALL	2,96	(0,58)	3,73	(1,27)	1,69	(0,20)
	MSSA	3,33	(0,49)	4,26	(1,03)	1,64	(0,22)
	MRSA	2,58	(0,37)	3,20	(1,26)	1,73	(0,18)
RM	ALL	2,56	(0,56)	1,80	(0,79)	0,74	(0,30)
	MSSA	2,92	(0,51)	2,12	(0,53)	0,84	(0,35)
	MRSA	2,21	(0,33)	1,49	(0,87)	0,64	(0,19)

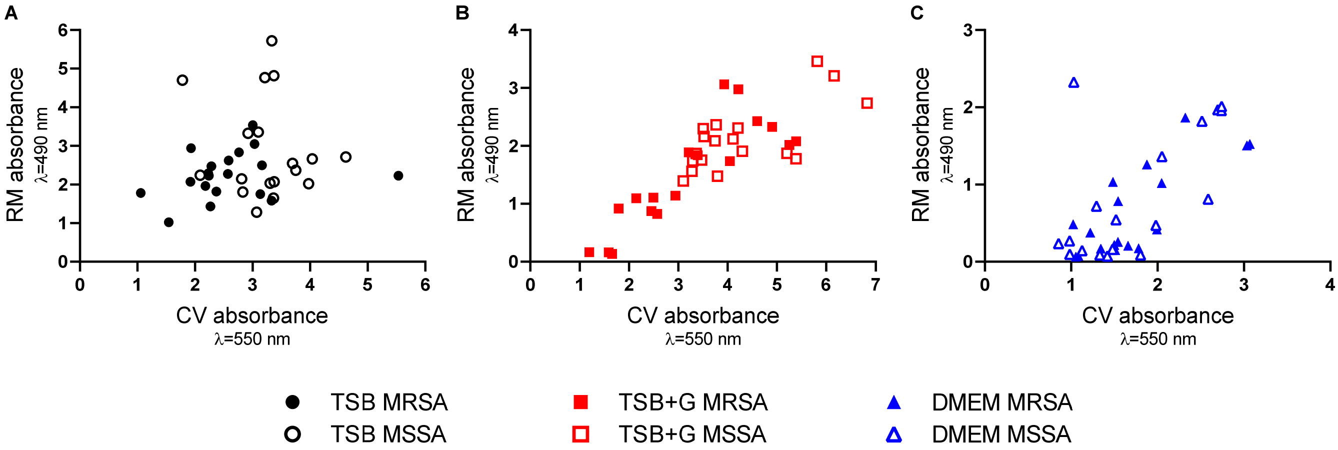

Figure S1. Scatter plots of correlations between the ability to form biofilm assessed with the Crystal Violet method (**CV**) and bacterial metabolic activity measured with the Richard's method (**RM**) for methicillin-susceptible *Staphylococcus aureus* **MSSA** and methicillin-resistant *Staphylococcus aureus* **MRSA** strains cultured in tryptic soy broth **TSB** (a), tryptic soy broth with 1% glucose **TSB+G** (b) and Dulbecco's Modified Eagle Medium **DMEM** (c).

Table S2. Zones of inhibitions of gentamycin **GENTA**, ciprofloxacin **CIPRO**, levofloxacin **LEVO** obtained by disk-diffusion method, and minimal inhibitory concentrations of vancomycin **VANCO** obtained by a strip diffusion method for methicillin-susceptible (S1-S5, ATCC 6538) and methicillin-resistant *Staphylococcus aureus* strains (R1-R5, ATCC 33591). Results were interprated following European Committee on Antimicrobial Susceptibility Testing breaking points table. S – susceptible, I – susceptible, increased exposure, R – resistant; ATCC – American Type Culture Collection.

Agar diffusion method	Zones of inhibition [mm]						MIC [mg/L]	
STRAIN	GENTA		CIPRO		LEVO		VANCO	
S1	30	S	32	I	31	I	0.5	S
S2	28	S	33	I	35	I	0.5	S
S3	27	S	32	I	33	I	1	S

**S4**	29	S	30	I	31	I	1	S
**S5**	26	S	25	I	33	I	2	S
**ATCC 6538**	26	S	28	I	32	I	1	S
**R1**	25	S	29	I	30	I	2	S
**R2**	25	S	0	R	0	R	0.5	S
**R3**	25	S	0	R	0	R	1	S
**R4**	0	R	11	R	18	R	2	S
**R5**	25	S	30	I	32	I	1.5	S
**ATCC 33591**	25	S	28	I	30	I	1	S

Table S3. Minimal Inhibitory Concentrations of gentamycin **GENTA**, ciprofloxacin **CIPRO**, levofloxacin **LEVO** vancomycin **VANCO** obtained by a microdilution method for methicillin susceptible (S1-S5, ATCC 6538) and methicillin resistant *Staphylococcus aureus* strains (R1-R5, ATCC 33591) cultivated in tryptic soy broth. Results were interpreted following European Committee on Antimicrobial Susceptibility Testing breaking points table. S – susceptible, I – susceptible, increased exposure, R – resistant; ATCC – American Type Culture Collection.

**Microdilution method**	**MIC [mg/L]**							
**STRAIN**	**GENTA**		**CIPRO**		**LEVO**		**VANCO**	
**S1**	0.98	I	0.49	I	0.15	I	1.22	S
**S2**	3.91	R	0.49	I	0.31	I	1.22	S
**S3**	1.95	R	0.49	I	0.15	I	1.22	S
**S4**	1.95	R	0.49	I	0.15	I	0.61	S
**S5**	1.95	R	0.24	I	0.08	I	0.61	S
**ATCC 6538**	1.95	R	0.24	I	0.15	I	1.22	S
**R1**	0.98	I	0.06	I	0.15	I	0.61	S
**R2**	1.95	R	R	R	19.53	R	2.44	R
**R3**	R	R	R	R	19.53	R	1.22	S
**R4**	3.91	R	7.81	R	4.88	R	1.22	S
**R5**	1.95	R	0.24	I	0.61	I	1.22	S
**ATCC 33591**	3.91	R	0.24	I	0.15	I	1.22	S

Table S4. Minimal Biofilm Eradication Concentrations of gentamycin **GENTA**, ciprofloxacin **CIPRO**, levofloxacin **LEVO** vancomycin **VANCO** obtained by a microdilution method for methicillin-susceptible (S1-S5, ATCC 6538) and methicillin-resistant *Staphylococcus aureus* strains (R1-R5, ATCC 33591). Results are presented in mg/L, strains for which the highest tested antibiotic concentration was insufficient to determine the MBEC values are marked as "-".

**STRAIN**	**GENTA**			**CIPRO**			**LEVO**			**VANCO**		
	**TSB**	**TSB+G**	**DMEM**	**TSB**	**TSB+G**	**DMEM**	**TSB**	**TSB+G**	**DMEM**	**TSB**	**TSB+G**	**DMEM**
**S1**	-	-	-	-	-	2	-	-	-	-	-	-
**S2**	-	-	2	-	-	1	-	-	-	-	-	-
**S3**	-	-	-	-	-	2	-	-	-	-	-	-
**S4**	-	-	0.0156	-	-	1	-	-	2.5	-	-	-
**S5**	-	-	-	-	-	-	-	-	-	-	-	-
**ATCC 6538**	-	-	2	-	-	2	-	5	-	-	-	-
**R1**	-	-	2	-	-	2	-	-	5	-	-	-
**R2**	-	-	-	-	-	-	-	-	-	-	-	-

**R3**	-	-	2	-	-	2	-	-	-	-	-	-
**R4**	-	-	2	-	-	2	-	5	0.3125	-	5	0.0195
**R5**	-	2	-	-	-	-	-	-	-	-	-	-
**ATCC 33591**	-	-	-	-	-	-	-	-	-	-	-	-
